# The Repertoire of RNA Modifications Orchestrates a Plethora of Cellular Responses

**DOI:** 10.3390/ijms24032387

**Published:** 2023-01-25

**Authors:** Panagiotis G. Adamopoulos, Konstantina Athanasopoulou, Glykeria N. Daneva, Andreas Scorilas

**Affiliations:** Department of Biochemistry and Molecular Biology, Faculty of Biology, National and Kapodistrian University of Athens, 15701 Athens, Greece

**Keywords:** post-transcriptional modifications, mRNA stability, translation efficiency, alternative splicing, writers, erasers, readers, m^6^A, m^5^C, Ψ

## Abstract

Although a plethora of DNA modifications have been extensively investigated in the last decade, recent breakthroughs in molecular biology, including high throughput sequencing techniques, have enabled the identification of post-transcriptional marks that decorate RNAs; hence, epitranscriptomics has arisen. This recent scientific field aims to decode the regulatory layer of the transcriptome and set the ground for the detection of modifications in ribose nucleotides. Until now, more than 170 RNA modifications have been reported in diverse types of RNA that contribute to various biological processes, such as RNA biogenesis, stability, and transcriptional and translational accuracy. However, dysfunctions in the RNA-modifying enzymes that regulate their dynamic level can lead to human diseases and cancer. The present review aims to highlight the epitranscriptomic landscape in human RNAs and match the catalytic proteins with the deposition or deletion of a specific mark. In the current review, the most abundant RNA modifications, such as N6-methyladenosine (m^6^A), N5-methylcytosine (m^5^C), pseudouridine (Ψ) and inosine (I), are thoroughly described, their functional and regulatory roles are discussed and their contributions to cellular homeostasis are stated. Ultimately, the involvement of the RNA modifications and their writers, erasers, and readers in human diseases and cancer is also discussed.

## 1. Introduction

Ever since the central dogma of molecular biology was proposed by Francis Crick, our perception of the flow of genetic information has been dramatically broadened. Over the years, the flux of information has revealed numerous processing steps that ensure proper gene expression and has highlighted the high complexity of the human genome, paving the way for deciphering the hidden aspects of life [1]. Moreover, the identification of various transcription factors and the functional clarification of the transcriptional machinery, as well as the rise of genomics, has provided new insights into the genetic programs that govern human development [2]. Although a plethora of chemical DNA modifications had been reported decades before the sequencing of the first nucleic acid, recent breakthroughs in sequencing techniques has enabled the in-depth study of genomes and has led to the introduction of epigenomics [3]. In the same manner, through the rise of epitranscriptomics, RNA sequencing attempts to decode the regulatory layer that rests between genome and proteome, namely transcriptome, and set the ground for the detection of modifications in ribose nucleotides [4,5].

**Figure 1:**
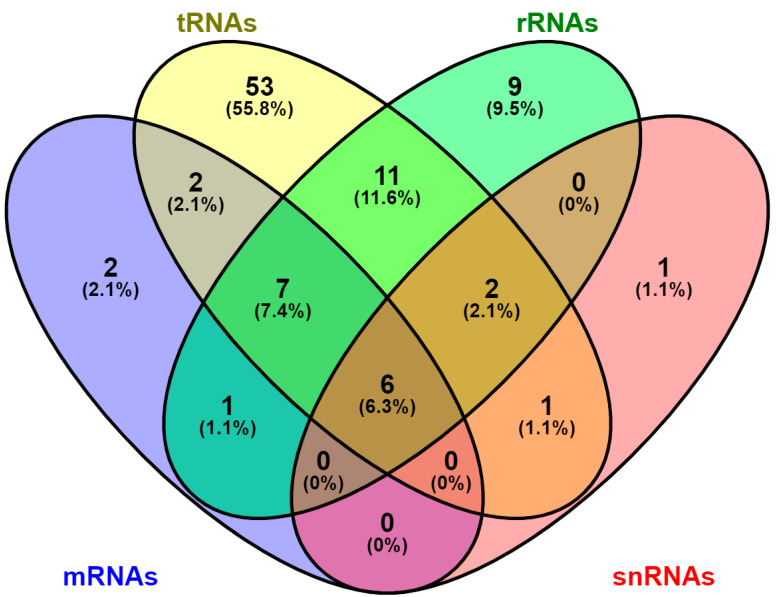
Venn diagram demonstrating the number of common RNA modifications that exist among many classes of RNA molecules, including tRNAs, mRNAs, and rRNAs, as well as snRNAs.

For decades, it was known that RNA molecules were decorated with various chemical modifications, but it was only after the revolution in molecular biology and the advent of RNA sequencing techniques that the emerging field of epitranscriptomics spawned. Until now, more than hundreds of multiple RNA chemical modifications have been reported, whereas only a few of them have been systematically studied [6] (Figure 1). These post-transcriptional changes have been observed in a wide spectrum of newly transcribed RNAs, including transfer RNAs (tRNAs), messenger RNAs (mRNAs), ribosomal RNAs (rRNAs), small non-coding RNAs, circular RNAs (circRNAs) and long non-coding RNAs (lncRNAs) (Supplementary Table S1).

RNA modifications are implicated in the regulation of several facets of RNA processing, thus possessing an indispensable role for the generation of functional RNA molecules [7,8]. These changes can be added or removed by different types of enzymes that catalyze their biosynthetic reactions. More precisely, the deposition of the chemical marks onto the RNAs is mediated by “writers”, a protein family that forms multiprotein complexes. The “writers” constitute a class of enzymes that act on RNAs and covalently introduce methyl and/or acetyl groups into the RNA nucleotides [9,10]. On the contrary, “erasers” represent enzymes that remove these epitranscriptomic marks, whereas “readers” are binding proteins responsible for the selective recognition of RNA modifications [11]. Notably, each modification is deposited, erased, or read by different members of the respective protein group that causes the alteration.

Interestingly, N6-methyladenosine (m^6^A), N5-methylcytosine (m^5^C), pseudouridine (Ψ) and inosine (I) are the most abundant RNA modifications in eukaryotic cells and their roles have been extensively investigated. Additionally, 7-methylguanosine (m^7^G), N1-methyladenosine (m^1^A), 3-methylcytidine (m^3^C), queuosine (Q), uridine (U) and ribose methylation (2′-O-Me or Nm) are RNA-related chemical marks that also fall under the scope of modern epitranscriptomics research (Supplementary Table S1) [6]. The present review aims to depict the human epitranscriptomic landscape in various types of RNA molecules and match the different RNA-modifying proteins with a specific mark. An additional goal is to summarize the functional and regulatory roles of each modification, among the different types of RNAs, in order to elucidate their contribution to cellular homeostasis. Ultimately, a thorough investigation of their involvement in human diseases, including cancer, is also discussed.

## 2. The Landscape of RNA Modifications

The widespread post-transcriptional process of RNA editing affects all nucleotides: A, U, C, and G. Of note, RNA modifications have been reported in all RNA types, including coding and non-coding ones. The abundancy of each modification among the different RNA species varies and depends on the phase of the cell cycle, the cell type, as well as the cellular responses and requirements. Additionally, it should be noted that several RNA modifications (e.g., m^1^A, I) can affect the Watson–Crick base-pairing, leading to either the misincorporation of nucleotides in the cDNA synthesis procedure or a blockage in the reverse transcriptase (Table 1). Consequently, these chemical marks induce ‘modified base pairs’ that impact on the RNA folding, 3D structures and flexibility of the molecules [12].

### 2.1. Chemical Modifications of Adenine RNA Base

Adenine, the most heavily modified RNA nucleotide, can be altered in different ways; this includes, for example, methylations on the nitrogen atoms of the adenine base generating m^6^A and m^1^A modifications, as well as conversions of adenine to inosine (A-to-I).

#### 2.1.1. N6-Methyladenosine (m^6^A)

Although it has been over 40 years since m^6^A was discovered, it is still the center of attention; many studies aim to investigate this type of RNA modification and its functionality, since it constitutes the most prevalent internal RNA change both in yeast and higher eukaryotes [13]. Going back to 1975, different research teams found that approximately 0.3% of total adenine bases are N6-methyl-modified [14,15], indicating its high abundancy in the transcriptome. Recent studies have confirmed that more than 8000 human mRNAs and more than 300 ncRNAs harbor m^6^A sites [16]. More precisely, Meyer et al. identified >40,000 m^6^A peaks in human RNAs, while their bioinformatics analysis supported that ~95% of the m^6^A are located at mRNAs, whereas <2% of the m^6^A sites are found in ncRNAs [16]. In human cells, m^6^A is in abundance on mRNAs, highlighting its involvement in multiple biological processes, whereas its presence in rRNAs affects translation. It should be noted that m^6^A methylation has also been detected in other RNA species, such as microRNAs (miRNAs) and circRNAs, contributing to their biogenesis and maturation; meanwhile, in lncRNAs m^6^A, bases are responsible for mediating transcriptional repression, thus affecting their functionality (Supplementary Table S1) [17]. On the contrary, although several epitranscriptomic studies focusing on tRNAs support the existence of a plethora of modifications, there is no evidence of m^6^A in the nucleotide sequence of the human tRNAs.

**Figure 2:**
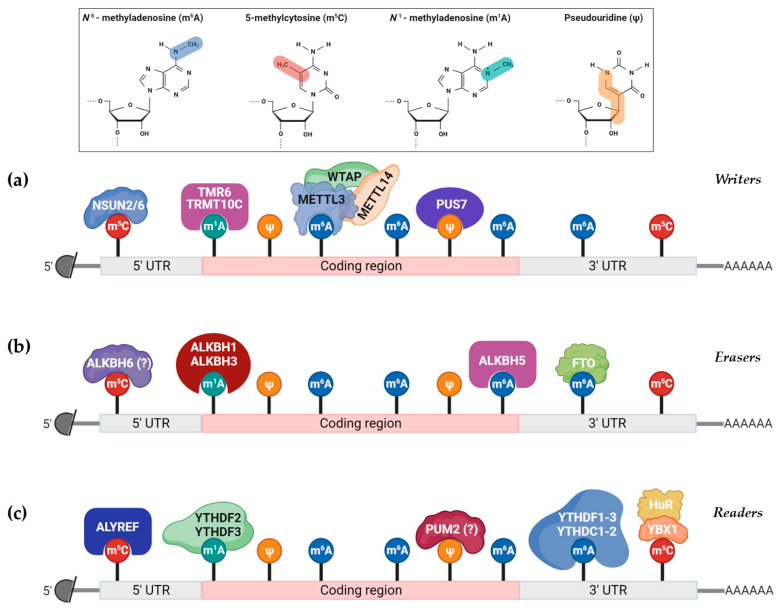
Common eukaryotic mRNA modifications that have been identified. The abundancy of each modification is strictly regulated by (**a**) “writers”, proteins that incorporate the specific modification in specific mRNA sites, (**b**) “erasers”, which catalyze the removal of a specific modification from the mRNA and (**c**) “readers”, which interact with specific modifications to regulate a wide spectrum of cellular processes.

The deposition of a methyl group onto the sixth nitrogen atom of A is catalyzed by specific enzymes, called m^6^A-methyltransferases [11]. More precisely, Methyltransferase-like 3 (METTL3) is the first confirmed methyltransferase that can individually incorporate m^6^A modifications into mRNAs and lncRNAs [18]. In the same manner, the high homolog of METTL3, Methyltransferase-like 14 (METTL14), catalyzes the formation of m^6^A (Figure 2). Multiple studies have reported that both METTL3 and METTL14 exhibit catalytic functions by themselves, indicating that they constitute the most significant m^6^A writers [19].

However, these enzymes form a multi-subunit complex that mediates the generation of m^6^A sites, synergistically targeting specific sequences known as DRACH motifs (D = G/A/U, R = G/A, H = A/U/C). Additional proteins, such as the Wilms tumor 1-associated protein (WTAP), the Vir-like m^6^A methyltransferase-associated (VIRMA) protein, the RNA binding motif protein 15/15B (RBM15/15B), and the Zinc-finger CCCH-type-containing 13 (ZC3H13) protein, are implicated in the formation and regulation of the m^6^A methyltransferase complex [20,21]. For instance, WTAP stabilizes the methyltransferase complex, whereas VIRMA guides the complex near the 3′ untranslated regions (UTRs) and the stop codon regions of the mRNAs [22,23,24]. Furthermore, except from these fundamental methyltransferases, it has been recently verified that an additional member of the protein family, Methyltransferase-like 16 (METTL16), can act in an independent manner, catalyzing the m^6^A formation in both mRNAs and lncRNAs [25,26].

In the case of miRNAs, various scientific reports have supported that pri-miRNAs can be methylated by either METTL3 or METTL14, which facilitate miRNA maturation [27,28]. In the same manner, METTL3 regulates m^6^A levels in circRNAs [29]. Interestingly, although limited information is known about the enzymes that are responsible for base modifications on human rRNAs, it has been proven that Methyltransferase-like 5 (METTL5) is the m^6^A “writer” of the 18S rRNA, which is stabilized by TRMT112, whereas ZCCHC4 acts on the 28S rRNA (Table 1) [30,31,32].

On the other hand, only two demethylases have emerged as the universal molecular “erasers” of m^6^A, the fat mass and obesity-associated protein (FTO), and the Human AlkB homolog H5 (ALKBH5) protein [33,34]; meanwhile, various binding proteins can read m^6^A alterations, including members of the YT521-B homology domain-containing proteins (YTHDF1-3 and YTHDC1-2), insulin-like growth factor 2 (IGF2), mRNA-binding proteins (IGF2BP1-3), Fragile X messenger ribonucleoprotein 1 (FMR1), Leucine-rich PPR motif-containing protein (LRPPRC), heterogeneous nuclear ribonucleoprotein (HNRNP) protein family members (HNRNPC and HNRNPA2B1) and the eukaryotic initiation factor 3 (eIF3) [10,11,13,35].

Undoubtedly, the existence of m^6^A in multiple RNA types manifests its involvement in the regulation of almost all phases of RNA metabolism, including RNA structure, localization, stability, and shelf life [21]. In human rRNAs, two m^6^A sites have been reported: one at position A1832 in 18S rRNA, as well as one at position A4220 in 28S rRNA (Table 2 and Table 3) [32,36,37]. Structural analyses have revealed that these RNA modifications are located at catalytic ribosomal regions and, therefore, may affect the function of the ribosomes being involved in different processes, such as ribosome heterogeneity, and translation rate and efficiency [38].

Recently, multiple studies have focused on the functional role of m^6^A modification in mRNAs, and its involvement in the mRNA metabolism and fate. m^6^A sites have been detected in various sites across the mRNAs of eukaryotic cells, such as the 5′ and 3′ UTRs, and the coding sequence (CDS); however, it is particularly enriched in 3′-UTRs around the termination codons. In particular, approximately 50% of the m^6^A sites are located at the last exon of the transcripts [16]. In addition, m^6^A modifications have been observed near exonic splice junctions in the CDS region and have been shown to affect the alternative splicing of human precursor mRNAs. More precisely, the mechanism involves the binding of the nuclear reader YTHDC1 to m^6^A sites, which recruits multiple splicing factors, including the SRSF3 that enhances exon inclusion and regulates the mRNA splicing by directly guiding the spliceosome in the appropriate splice sites [13,39].

The nuclear export of mature transcripts is catalyzed by writers, erasers, and readers of m^6^A. Specifically, m^6^A hypermethylated sites facilitate the transportation of the mRNA into the cytosol by their binding to the nuclear protein YTHDC1, which delivers the molecule to nuclear export proteins [40]. Furthermore, into the cytosol, the m^6^A methylation regulates protein synthesis through a plethora of enzymes that cooperate to increase translation efficiency [41,42]. METTL3 incorporates m^6^A into the UTRs and binds to eIF3 to form the mRNA loop and promote translation, whereas YTHDF1 and YTHDF3 can also enhance translation through the YTHDF-eIF3 pathway [43,44]. Notably, the recruitment of m^6^A readers is also necessary for maintaining mRNA stability, while m^6^A sites regulate secondary structures [45]. Associated studies have supported that, in human cells, the downregulation of the writer protein METTL3 has led to an increase in the half-life of the mRNAs, indicating that m^6^A levels directly affect the mRNA’s fate [46].

#### 2.1.2. N1-Methyladenosine (m^1^A)

**Figure 3:**
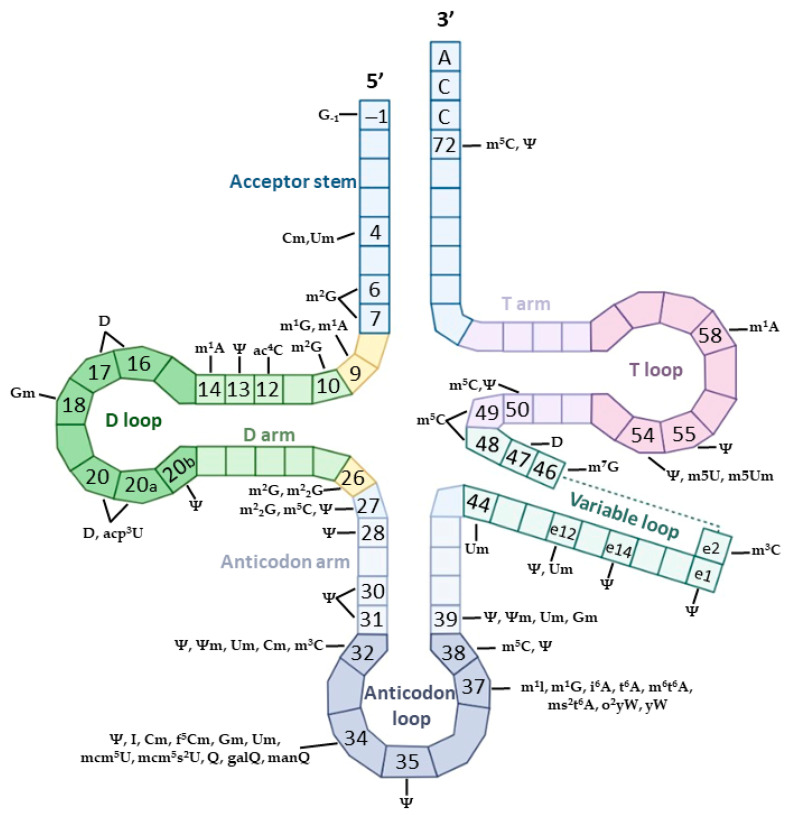
Graphical representation of the identified nucleotide modifications in eukaryotic tRNAs and their corresponding position in the tRNA sequence.

The m^1^A constitutes an additional methylation of adenine and the m^1^A RNA-modifying proteins regulate its dynamic abundancy in both ncRNAs and mRNAs. Most m^1^A sites have been identified at specific positions in tRNAs, around the GC-rich regions, (Figure 3), whereas m^1^A is conserved in position 1309 of the human 28S rRNA (Table 3).

M^1^A has also been found in specific sites in mitochondrial RNA (mt-RNA), as well as in HGGAGRA motifs ((H = A/U/C, R = G/A) of the lncRNAs [47]. In the case of mRNAs, studies have shown that only one m^1^A modification is present in each transcript within the GUUCNANNC sequence, which is mainly located in the 5′ UTRs and/or in the first splice site [48,49]. In contrast to m^6^A, the addition of a methyl group into the first atom of the base is catalyzed by TRMT10 and the TRMT6/TRMT61 complex, which are members of the tRNA methyltransferase protein family (TRMT); meanwhile, ALKBH1 and ALKBH3 are the key enzymes that erase this type of modification (Table 1). Notably, Nucleomethylin (NML), an additional methylase, introduces methylations on 28S rRNA [50,51]. As for the proteins that recognize m^1^A sites, it has been shown that YTHDF2 and YTHDF3 have a low binding affinity for m^1^A. Although Heat-responsive protein 12 (HRSP12) is characterized as a factor involved in the RNase P/MRP-mediated endoribonucleolytic cleavage of m^6^A, a recent report supports that HRSP12 functions as a reader protein for m^1^A [52].

To continue, m^1^A methylations may play a regulatory or stabilizing role in modified RNAs. As for the tRNA molecules, m^1^A in position 58 stabilizes the T-loop structure. Especially in tRNAiMet, the m^1^A58 increases translation by activating polysomes [53]. On the contrary, it is known that the overexpression of m^1^A erasers decreases the translation levels, while in the case of ALKBH3 overexpression, tRNA fragments (tRFs) can be formed through tRNA cleavage [54]. In rRNAs, m^1^A participates in the formation of the 60S subunit of the 80S complex [55]. Although m^1^A alterations in 5′ UTRs of the mRNAs enhance translation efficiency, m^1^A in the CDS prevents protein synthesis [56]. Finally, the m^1^A YTHDF2 and YTHDF3 proteins mediate the mRNA destabilization [50,57].

#### 2.1.3. Adenine to Inosine (A-to-I)

In humans, the most common RNA editing procedure that results in the substitution of a specific nucleotide is adenine-to-inosine (A-to-I); in mRNA, this is catalyzed by adenosine deaminase enzymes, namely ADARs, that act on double-stranded RNAs (dsRNAs). The ADAR family includes three proteins, which are encoded by three ADAR genes [58]. The typical protein structure of ADARs includes two distinct domains: the dsRNA binding domain in the N-terminal and the deaminase domain in the C-terminal. More precisely, ADAR1 and ADAR2 are globally expressed, possess a well-characterized adenosine deamination activity and typically act on pre-mRNAs; however, in the case of ADAR3, which is expressed in brain tissues, no deamination activity has been reported and thus its function remains unclear (Table 1) [59]. However, recent studies have connected ADAR3 with RNA editing inhibition, suggesting that ADAR3 acts as a negative regulator of A-to-I editing [60,61]. Especially on tRNAs, the formation of inosine at position 34 is catalyzed by the ADAT2/ADAT3 complex (Figure 3) [62,63]. 

Recent RNA-seq studies have confirmed a great number of A-to-I RNA editing sites in human mRNAs. Interestingly, the majority of inosine residues are located at UTRs and intronic regions; meanwhile, approximately 1000 editing sites have been found in protein-coding regions, indicating that A-to-I editing has two distinct and critical roles in RNAs [58,64,65]. Firstly, I bases affect the mRNA’s structure and influence the binding affinity of proteins to the mRNA. Additionally, splicing and translation machineries recognize G instead of I, influencing both splicing patterns and translation accuracy [66]. Secondly, I_34_ of tRNA is located at the wobble position and has been related to the codon recognition mechanism [62,63]. Taken together, A-to-I editing constitutes a significant modification that mediates protein synthesis in different layers, giving birth to various proteins that, therefore, increase the proteome diversity.

### 2.2. Chemical Modifications of Cytosine RNA Base

Apart from adenine, a great number of post-transcriptional marks have also been found in cytosine, among which m^5^C, m^3^C methylations, C-to-U RNA editing and the synthesis of pseudouridine and dihydrouridine participate in various biological processes; thus, these are at the center of epitranscriptome research.

#### 2.2.1. N5-Methylcytosine (m^5^C)

The most dominant methylation of C in RNAs is the one occurring in the fifth nitrogen atom of the C, namely m^5^C. This type of modification has been found in diverse types of RNAs. Two distinct methyltransferase groups have been confirmed to incorporate m^5^C in RNAs: the NOP2/Sun RNA methyltransferase (NSUN) family that includes seven proteins and the DNMT2 [67]. On the contrary, Ten-eleven translocation methylcytosine dioxygenase 1 (TET1) is a recently identified m^5^C eraser, whereas TET2 and TET3, MBD2/4, ALKBH1 and ALKBH6 are potential m^5^C demethylases; however, none of them have been confirmed [68,69,70,71,72]. Furthermore, the Aly/REF export factor (ALYREF), Y-box binding protein 1 (Ybx1) and the Radiation sensitive 52 (RAD52) have been identified as the m^5^C recognition proteins [73,74,75,76,77].

To begin with, m^5^C is present at multiple positions in human tRNAs, including C38, C48 and C72, which are modified by NSUN2, NSUN6 and DNMT2 (Figure 3) [67]. In particular, the incorporation of the methyl group at C38 in the anticodon loop is catalyzed by DNMT2. Studies have reported that DNMT2 prevents tRNA from degradation and hence m^5^C38 enhances translation. Methylation in C48 facilitates the binding of m^5^C38 with nucleoside at position 15, forming the “Levitt pair”. This bond leads to the generation of the characteristic L-structure that stabilizes the molecule. Additionally, C72 in the acceptor stem is critical for tRNA biogenesis and is methylated by NSUN6, whereas the 3′-CCA motif is required [78]. The m^5^C modifications on tRNAs ensure the increased stability of the molecules and their appropriate folding, and facilitate codon–anticodon interactions [79,80].

In human rRNAs, m^5^C methylations have only been detected in the 28S rRNA and incorporated by two NSUN proteins, NSUN1 and NSUN5. NSUN1 regulates transcription and targets the C4417 residue, whereas NSUN5 binds to the position 3761 of the 28S rRNA [81,82]. Both modified nucleotides support the translational fidelity and the proper folding of the 28S rRNA. Furthermore, NSUN4 introduces a methyl group into the mt-12S rRNA at position C841 [83]. m^5^C is also found in lncRNAs, where it facilitates its biogenesis and ensures its stability [84]. Several studies report NSUN7 as the potential writer of m^5^C in ncRNAs [78,85].

For more than 60 years, it has been known that m^5^C decorates mRNAs, but the exact locations of this mark were unknown until the advent of liquid chromatography–mass spectrometry and next-generation sequencing, which enabled the mapping of the m^5^C in single nucleotide resolution [77]. Relevant studies support that UTRs are rich in m^5^C, while in CDS regions, the m^5^C sites are depleted [73,86]. Until now, NSUN2 is the only confirmed m^5^C mRNA writer, whereas ALYREF serves as the reader protein. Remarkably, the m^5^C status regulates a plethora of cellular responses that affect mRNA fate and its exportation from the nucleus. The wide distribution of m^5^C within the mRNAs affects the translation efficiency in multiple ways. Firstly, the enriched m^5^C sites in 5′ UTRs modulate the protein translation, while during cell aging, erasers are activated to demethylate these sites. Additionally, the increased accumulation of m^5^C modifications in the 3′ UTRs demonstrates an increased translational capacity [78,87]. As for the internal mRNA regions that are subjected to m^5^C methylations, m^5^C can reduce translation efficiency by altering the codon–anticodon binding affinity [88]. Overall, m^5^C in mRNAs are associated with vital biological processes, including nuclear–cytoplasmic shuttling, maternal mRNA stabilization, splicing and the translation rate [89].

#### 2.2.2. N3-Methylcytidine (m^3^C)

The m^3^C represents a tRNA modification, which is found in position 32 in different species [90,91]; however, in some human tRNAs, it is also located at the e2 position of the variable loop (Figure 3). Additional reports have mentioned the presence of m^3^C on mRNAs, but in much lower levels [92]. Although it was known that TRM140 methyltransferase inserts m^3^C in tRNAs of *Saccharomyces cerevisiae*, METTL2A, METTL2B, METTL6, and METTL8 have recently been confirmed as human m^3^C methyltransferases [93,94]. Notably, METTL2A, METTL2B and METTL6 act on tRNAs, whereas METTL8 methylates sites on mRNAs.

On the contrary, the human ALKBH3 erases m^3^C on tRNAs, but demethylation on mRNAs is achieved by ALKBH1 [95,96]. As the molecular role of m3C32 in tRNAs, it has been shown to interact with the nucleotide at position 38, which leads to the maintenance of the anti-codon loop structure and the increase in the decoding accuracy. Due to its low abundancy, the functionality of m^3^C in mRNAs remains unclear [97].

#### 2.2.3. Cytidine to Uridine (C-to-U)

Except from A-to-I, another RNA editing mechanism that involves the conversion of bases is the formation of U by C. The Apolipoprotein B mRNA editing enzyme (APOBEC) converts the ribobase C to U in mRNA editing sites [98]. Although C-to-U editing has been suggested to be involved in mRNA stability and translation accuracy, there are no relative studies to prove that statement.

#### 2.2.4. Pseudouridine (Ψ)

The Ψ modification is the well-studied derivative of U and is prevalent in all classes of RNAs. In humans, Ψ is mainly formed by members of the pseudouridine synthase (PUS) family, PUS1-PUS10, while its readers and erasers remain uncertain. Ψ writers can be divided into two separate groups: the guide RNA-dependent synthases that include the small ribonucleoproteins H/ACA sRNPs, and the guide-independent PUS enzymes. Briefly, the guide-dependent process is catalyzed by a two-step reaction that requires a complementary RNA to guide the enzyme in the target region and a protein that forms the modification [99]. On the contrary, the guide-independent pathway utilizes the members of the PUS family, which can directly recognize the target sites [100]. However, the RNA-binding protein PUM2 can recognize the UGUAR motif in human cells and, therefore, is a candidate for being a potential Ψ reader [101,102]. Undoubtedly, the high levels of Ψ in all the types of RNA reflect on its multidimensional implications in the RNAs’ life. Notably, the great thermodynamic stability of the Ψ-modified RNAs is based on the strength of the bond that is created between Ψ and A, whereas Ψ can also stabilize single-stranded RNAs. In tRNAs, Ψ affects a plethora of cellular responses, including tRNA biogenesis, degradation, and the production of tRFs. In particular, Ψ39 in the anticodon arm increases the melting temperature, controlling the tRNA folding process [103]. 

**Figure 4:**
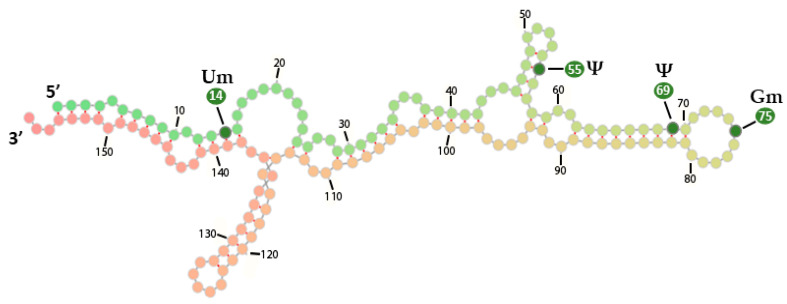
Schematic demonstration of the detected RNA modifications in human 5.8S RNA, as well as their respective positions in the RNA sequence.

rRNAs are also subjected to pseudouridylation through Dyskerin Pseudouridine Synthase 1 (DKC1), an alternative enzyme that incorporates Ψ in 28S rRNAs [104]. Notably, although an increased number of Ψ sites had already been detected, seven additional Ψ residues have recently been identified in significant ribosomal regions: the first four at the positions 897, 1045, 1136, 1232 of the 18S rRNA and the rest at positions 1768, 2619 and 4463 of the 28S (Table 2 and Table 3). The 5.8S rRNA is also subjected to Ψ modifications at specific locations (Figure 4). The high density of Ψ in rRNAs underlines its importance in ribosome assembly and translational fidelity [105].

In mRNAs, Ψ is added co-transcriptionally by PUS1, PUS7, and RPUSD4 enzymes, and plays a critical role in the alternative pre-mRNA processing steps that affect gene expression [105,106,107]. Moreover, Ψ sites are distributed throughout mRNA sequences, being present in UTRs and CDS regions, and their regulatory role is to control mRNA metabolism [105]. The mapping of Ψ sites uncovered that human mRNAs are highly modified, ranging from 10–50% [108]. Briefly, Ψ can alter the primary protein sequence through the misincorporation of one or more amino acids, or even promote the termination codon readthrough during protein synthesis [109]. Furthermore, it should be mentioned that snRNAs harbor a plethora of Ψ sites crucial for their interactions with other RNAs and protein molecules. For instance, Ψ6, Ψ7 and Ψ15 in the U2 snRNA sequence are necessary for the assembly of the spliceosome machinery [110,111]. In the same manner, similar modifications in U4, U5 and U6 snRNAs enhance the mechanism of splicing [112].

#### 2.2.5. Dihydrouridine (D)

Besides Ψ, C can be edited into U to synthesize D, a highly conserved alteration found in great abundancy at specific positions in the D-loop of tRNAs [113]. Dihydrouridine synthase (DUS) enzymes are four protein molecules that form D in human tRNAs, with DUS2 being the most dominant one. The incorporation of D into tRNAs has been linked to tRNA folding and the increased flexibility of tRNAs, and it is possible to destabilize their 3D structures [114,115]. Although D was determined to be a tRNA-specific modification, recent sequencing-based studies have supported the existence of D in coding RNAs and lncRNAs; however, its biological role is still ambiguous [116].

### 2.3. Chemical Modifications of Guanine RNA Base

Even though modern epitranscriptomics is mainly focused on modifications occurring in adenine and cytosine, several guanine-based alterations are vital for the fate of RNAs and are thus worth mentioning.

#### 2.3.1. N7-Methylguanosine (m^7^G)

Although the m^7^G mark is widely known due to its presence in tRNAs originating from multiple organisms, it is also associated with the eukaryotic 5′ capping of the mRNA [117,118]. Different methyltransferase complexes act in the RNAs to incorporate this type of modification. Specifically, in humans, the METTL1/WDR4 complex participates in the formation of m^7^G in tRNAs, whereas WBSCR22 and TRMT112 proteins act synergistically to add m^7^G on the 18S rRNA [119,120,121]. METTL1 can also methylate miRNAs, such as let-7 miRNA, a critical procedure for their biogenesis [122]. On the other hand, RNA guanine-7 methyltransferase (RNMT) is responsible for the m^7^G addition to the mRNA cap [47].

As for the physiological role of m^7^G in tRNAs, the formation of m^7^G46 promotes the mRNA translation and increases the tRNA stability [117]. It should be noted that, although the catalytic activity of WBSCR22 recruits the m^7^G1639 in the human 18S rRNA, this modification is not necessary for the biogenesis of the 40S ribosomal subunit [120]. Moreover, the special features of m^7^G in the 5′ cap of the mRNA enable its involvement in vital biological pathways, including RNA maturation, nuclear export and cap-dependent translation [118].

#### 2.3.2. N1-Methylguanosine (m^1^G)

In human RNAs, the addition of a methyl group into the first atom of the ribose ring of G is mediated by various writers that act independently. More precisely, tRNA methyltransferase 5 (TRMT5) is the enzyme that catalyzes the incorporation of m^1^G at tRNA position 37; meanwhile, in case of mitochondrial tRNAs, TRMT5 demonstrates a notably lower activity and is replaced by TRMT10C, an additional tRNA methyltransferase that introduces the m^1^G9 mt-tRNA modification [123,124]. Furthermore, the m^1^G9 methyltransferase TRMT10C can form multiprotein complexes with RG9MTD1 and SDR5C1, in order to catalyze the process of G methylation [125,126]. According to several studies, the formation of m^1^G in tRNAs is vital for proper tRNA folding and its tertiary structure, and can prevent frameshifting during protein production [127]. Moreover, as far as mRNA is concerned, although m^1^G writers have not been reported, the incorporation of a single m^1^G mark into the mRNA sequence destabilizes the translation machinery, leading to reduced levels of the generated protein [128].

#### 2.3.3. Queuosine (Q)

Q represents a tRNA-specific hyper-modified guanosine nucleoside derived from G, which forms a plethora of derivatives, including galactosyl-queuosine (GalQ), mannosyl-queuosine (ManQ), and glutamyl-queuosine (GluQ) [129,130]. It is worth mentioning that Q_34_ on tRNAs protects tRNAs from ribonuclease degradation and affects the translation accuracy [131,132].

### 2.4. Chemical Modifications of Uracil RNA Base

Although chemical modifications mainly occur in adenine, cytosine and guanine, few studies have reported the existence of post-transcriptional marks in U.

#### 2.4.1. N5-Methyluridine (m^5^U)

In RNAs, the methylation of the fifth nitrogen atom of the U ring creates the 5-methyluridine (m^5^U), which constitutes a common modification both in tRNAs and rRNAs. However, high-throughput sequencing studies have also revealed the existence of m^5^U in mRNAs. Although TRMT2A and TRMT2B are the m^5^U-catalyzing protein enzymes that add the methyl group into human tRNAs, rRNAs and mRNAs, neither erasers nor readers have been identified yet [133,134]. Of note, m^5^U regions have been extensively studied in human tRNAs and are found at position m^5^U54 in the T-loop, but also in the mitochondrial tRNAs [134]. Its function is to maintain and stabilize the tertiary structure of tRNAs; as a result the absence of the m^5^U54 mark can cause tRNA’s degradation and the generation of tRFs [135].

#### 2.4.2. N3-Methyluridine (m^3^U)

m^3^U is a major rRNA modification in multiple species, including humans, that is detected in human 28S rRNA at position 4500 (Table 3). Until now, although recent reports support that Beta-mannosyltransferases, Bmt5 and Bmt6, are responsible for the addition of methyl groups into the rRNA of *Saccharomyces cerevisiae*, the human m^3^U writer remains unknown [136]. Hence, its functional role in the rRNAs is unclear.

#### 2.4.3. Uridylation

In higher eukaryotes, the post-transcriptional addition of nucleotides in the 3′ UTRs is a major procedure for the stabilization of the newly synthesized RNA molecules [137]. The 3′ uridylation constitutes a widespread mechanism that is catalyzed by the terminal uridyltransferases TUT4 and TUT7 on different types of RNAs, in order to mark the molecules for degradation [138,139]. Notably, the TUT4/TUT7 complexes target mRNAs and miRNAs, and control both stability and RNA homeostasis by fine-tuning RNA levels during apoptosis [140]. An additional member of the TUT family, TUT1, has also been reported to catalyze uridylation, while DIS3 Like 3’-5’ Exoribonuclease 2 (DIS3L2) recognizes the uridylated sites [141].

### 2.5. 2′-O-Methylation (Nm) Modification

The Nm modification occurs post-transcriptionally via the incorporation of a methyl substituent into the 2′-hydroxyl of the ribose in any base. Consequently, the Am, Cm, Gm and Um modifications are generated. The Nm is widely distributed in all RNA types; however, tRNAs and rRNAs are particularly enriched in this type of alteration [142,143]. It has been shown that Nm affects the RNA structure by increasing the thermodynamic stability of the molecule in order to protect it from ribonucleases, and to enhance the RNA:RNA base pairing and the formation of RNA duplexes [144,145,146]. On the contrary, the tertiary RNA structures are disrupted, and the RNA–protein interactions are inhibited. 

In tRNAs, Nm marks are mainly deposited by FtsJ RNA 2’-O-Methyltransferase 1 (FTSJ1), which recognizes the C32 and N34 regions in the anticodon loop, and the creation of Cm32 and Nm34 influences the translation [147]. Additionally, TRMT44 constitutes a potential Um writer in tRNA^Ser^ [148]. In rRNAs, in the same manner as Ψ, Nm modifications are formed in pre-rRNAs and their role is to prevent hydrolysis and thus increase the structural rigidity of rRNAs [149]. Nm methylations are induced by snoRNAs that activate and guide Fibrillarin (FBL), the 2′-O-RNA methyltransferase, to the target rRNA, contributing to the fine-tuning of its function [143,150].

In mRNAs, Nm is present in the UTRs and more precisely in the 5′ mRNA termini, whereas in CDS regions, the AGUA motif has been found to harbor this type of modification [151]. The 5′ mRNA termini of eukaryotic organisms are heavily methylated and can form three types of caps: cap-0, cap-1 and cap-2. Nm is involved in the 5′ cap-1 (m7GpppNm) that is produced by CMTR1 and the 5′ cap-2 that includes the highly conserved m7GpppNmNm region, generated by CMTR2 [152]. The Nm modifications are highly involved in transcription processing, the mRNA stability and the protein synthesis efficiency [152].

## 3. RNA Modifications in Human Disease

Over the years, systematic efforts to catalog the repertoire of modifications that are embroidered on RNA molecules in different tissues and diverse pathophysiological conditions has illuminated the correlation between epitranscriptome deregulation and disease development (Table 4).

More specifically, mutations, as well as irregular expression patterns in numerous RNA modification enzymes, have been linked with defects in the epitranscriptome and, subsequently, with several human diseases, including cancer and neurological and cardiovascular disorders; thus, research has been propelled towards the elucidation of the enigmatic molecular mechanisms driving these pathological conditions [162].

First and foremost, a wide array of RNA modifiers have been associated with guiding normal cells towards the acquisition of traits distinctive of cancer cells, widely known as the “hallmarks of cancer” [171]. For instance, the m^6^A writer METTL3 has been found to be overexpressed in acute myeloid leukemia (AML) cell lines; it is considered to be accountable for the increased m^6^A methylation profiles and the translational activation of the MYC proto-oncogene, phosphatase and tensin homolog (PTEN), and the BCL2 apoptosis regulator mRNA transcripts, thus sustaining cell survival and proliferation [172]. On the other hand, in AML, the upregulated m^6^A eraser FTO contributes to the bypassing of growth suppressors by demethylating; this reduces the stability and negatively regulates the retinoic acid receptor alpha (RARA), the ankyrin repeat and SOCS box-containing 2 (ASB2) mRNAs, hampering cell differentiation and promoting leukemogenesis [173].

Notably, recent studies have also underlined the involvement of the m^6^A readers YTHDF1 and YTHDF2 in hindering the efficiency of immune responses against tumor antigens, and assisting leukemia stem cells to evade apoptosis, respectively. Specifically, YTHDF1 recognizes the m^6^A signature and enhances the translation of the lysosomal proteases’ mRNAs found in dendritic cells, which, in turn, degrade the engulfed antigens, inhibiting their cross-presentation and suppressing the induction of CD8+ T cell responses [174]. On the contrary, overexpressed YTHDF2 in AML cells spots m^6^A-methylated target transcripts, such as the tumor necrosis factor (TNF) receptor superfamily member 1B (TNFRSF1B) mRNAs, and promotes their degradation, affecting the TNF apoptotic signaling pathway [175].

Despite being the most frequently encountered RNA modification, m^6^A is not the only epitranscriptomic mark that can promote malignancy. In the cytoplasm, Lin-28 homolog A (*LIN28A*) normally regulates the expression of let-7 miRNAs, the recruitment of writer TUT4. In turn, TUT4 adds uridines at the 3′ end of the precursor miRNAs (pre-let7), thus inhibiting their cleavage by Dicer and promoting their degradation [176]. In breast cancer, however, an overexpression of LIN28A has been detected to lead to the deregulation of the aforementioned pathway and consequently the downregulation of the tumor-suppressing let-7 miRNAs [177]. Moreover, in chronic myeloid leukemia (CML), the activity of A-to-I writer ADAR1 has been proven to hinder let-7 miRNAs’ biogenesis, enhancing the replicative capacity of leukemia stem cells and promoting cancer progression [178]; meanwhile, in colon and lung cancer, the downregulation of the m^7^G writer METTL1 interferes with the methylation and maturation of the same miRNA family, resulting in the enhanced migration of cancer cells [122]. 

As far as the Ψ writer DKC1 is concerned, mutations in this gene have been linked with the reduced pseudouridylation of rRNA molecules, and subsequently with the modified translation of several cancer-associated transcripts, such as the vascular endothelial growth factor (VEGF) and the tumor suppressor p53 mRNAs [179,180,181]. On the other hand, the overexpression of NSUN2 in bladder carcinoma, and thus the m^5^C aberrant methylation of the oncogenic transcripts of the heparin binding growth factor (HDGF) gene, have been shown to augment mRNA stability and correspond with poor cancer prognosis [95]. Finally, the elevated expression of the eraser ALKBH3 and, therefore, the increased m^1^A demethylation of tRNA molecules, participate in the production of tRNA-derived small RNAs (tDRs) and contribute to an enhanced cancer cell proliferation [95]. 

Given its critical role in governing brain development and functionality, it is not surprising that perturbations in the m^6^A signature could also be implicated in numerous neurological diseases [182]. So far, several alterations in the m^6^A machinery have been detected, underlining the potentially critical role of defective methylation in the establishment and progression of these diseases [183]. Specifically, the FTO gene has already been correlated with neurodevelopmental and neuropsychiatric disorders, such as structural malformations and functional deficiencies of the brain, growth retardation, psychomotor retardation, attention-deficit/hyperactivity disorder (ADHD) and major depressive disorder (MDD) [184,185,186]; meanwhile, an increasing number of studies also highlight the link between this specific eraser and neurodegenerative disorders, such as Alzheimer’s disease (AD) [187,188,189], Parkinson’s disease (PD) [190,191] and amyotrophic lateral sclerosis (ALS) (Table 4) [192]. Furthermore, recent studies have reported the elevated expression levels of METTL3 in mouse models for AD [189], whereas mutations in the m^6^A eraser ALKBH5 and the m^6^A reader YTHDC2 have been shown to participate in MDD and autism spectrum disorder, respectively [193,194].

As for the remaining epitranscriptomic marks, alterations in the NSUN2 gene have been identified as a cause of developing autosomal-recessive intellectual disability (ID) [195] and Dubowitz syndrome (DS) [196], while mutations in PUS7 have been proven to result in the reduced pseudouridylation of specific sites in tRNA molecules, leading to microcephaly and ID [197]. Finally, increased Ψ marks seem to be linked with early-stage AD and myotonic dystrophy [198,199], whereas the reduced editing of AMPA and kainate glutamate receptors, due to the downregulated expression of ADAR2, has been correlated with the etiology of mental disorders such as bipolar disorder and schizophrenia [200].

Last but not least, RNA modifications are the key regulators in multiple cardiovascular diseases, including adipogenesis, obesity, type 2 diabetes, different types of atherosclerosis and limb ischemia (Table 4). In particular, the levels of m^6^A are responsible for the glucose metabolism by regulating the β-cells in the pancreas and urging liver gluconeogenesis, thus affecting the progression of type 2 diabetes. Both METTL3 and METTL14 are downregulated in patients with type 2 diabetes, whereas FTO demonstrates an increased expression pattern, which enhances the expression of forkhead box O1 (FoxO1), glucose-6-phosphatase (G6PC), and diacylglycerol acyltransferase 2 (DGAT2) enzymes; this finally leads to hyperglycemia, due to insulin secretion dysregulation. According to additional studies, an m^6^A-dependent pathway, in which FTO and METTL3 possess significant roles, controls lipid production and switches on/off both adipogenesis and obesity. 

In the case of atherosclerosis, the expression levels of m^6^A writers, readers and erasers can also promote and/or inhibit chronic inflammation and lipid deposition, which form the atherosclerotic plaques. For instance, the overexpression of METTL14 in endothelial cells is responsible for monocyte aggregation, via affecting either the m^6^A levels of FoxO1 mRNA or the binding affinity of the FoxO1 protein, resulting in the progression of atherosclerosis. The dysfunction of endothelial cells is also induced by the m^5^C RNA modification levels. Notably, NSUN2 can promote the translation of ICAM-1, which increases the adhesion of leukocytes to the surface of endothelial cells. On the contrary, limb ischemia is connected to other RNA modifications, such as A-to-I, Ψ and m^7^G. Studies support that A-to-I editing and Nm can alter the targets of the tumor suppressor microRNA, miR487b, and hence promote angiogenesis; meanwhile, the increased levels of the Ψ writers, RPUSD3 and RPUSD4, induce mitochondrial protein synthesis, leading to the defective assembly of OXPHOS [201]. Recent studies have pointed out that m^7^G modification in tRNAs is involved in vascular development due to the significant role of METTL1 in neovascularization [202]. Notably, m^7^G and the downregulation of METTL1 affect the pluripotency of the human-induced pluripotent stem cells, enable their differentiation to endothelial progenitor cells, and thus promote post-ischemic angiogenesis [203].

## 4. Conclusions

In summary, the rise of epitranscriptomics has led to the identification of a wide spectrum of RNA modifications that exist in many RNA classes. Each modification is located at specific regions in RNAs and possesses specific roles that are often vital to cellular responses. A variety of enzymes are implicated in the mechanisms that incorporate or erase each modification, thus underlining the complexity of the eukaryotic transcriptomes. The dysfunction of the catalytic enzymes that are responsible for the post-transcriptional marks into the RNAs can lead to human diseases, such as cancer. Modern epitranscriptomics aims to decipher the molecular mechanisms that generate the RNA modifications and decode the involvement of RNA marks in cellular homeostasis. Undoubtedly, breakthroughs in molecular biology, including RNA-sequencing techniques, will enhance our efforts to unveil the mysteries of the features of RNA molecules.

## Figures and Tables

**Table 1:** Enzymes catalyzing the addition, removal and recognition of common RNA modifications in human cells.

Modification	Base Pairing	Enzyme Type	Enzyme Name
m^6^A	Canonical	Writers	METTL3, METTL14, METTL16, METTL5, ZCCHC4
		Erasers	ALKBH5, FTO
		Readers	YTHD, IGF2, IGF2BP1-3, eIF3, FMR1, LRPPRC, HNRNPC, HNRNPA2B1
m^1^A	Non-canonical	Writers	TRM6, TRMT10C, TRM61A, TRM61B, NML
		Erasers	ALKBH1, ALKBH3
		Readers	YTHDF2?, YTHDF3
A-to-I	Non-canonical	Writers	ADAT2-ADAT3, ADAT1, ADAR2, ADAR1
		Erasers	DAP3?
		Readers	?
m^5^C	Canonical	Writers	NSUN1 to NSUN7, DNMT2, p120, TRDMT1
		Erasers	TET1 to TET3?, MBD2/4?, ALKBH1?, ALKBH6?
		Readers	ALYREF, YBX1, RAD52
m^3^C	Non-canonical	Writers	METTL2A, METTL2B, METTL6, METTL8
		Erasers	ALKBH1, ALKBH3
		Readers	?
m^1^G	Non-canonical	Writers	TRMT5, TRMT10, RG9MTD2, RG9MTD1, RG9MTD3, SDR5C1
		Erasers	?
		Readers	?
m^7^G	Canonical	Writers	WBSCR22/TRMT112, RNMT, WDR4, METTL1
		Erasers	?
		Readers	?
Q	Non-canonical	Writers	?
		Erasers	?
		Readers	?
C-to-U	Non-canonical	Writers	APOBEC1, APOBEC3A?, APOBEC3G?
		Erasers	?
		Readers	?
D	Non-canonical	Writers	DUS1 to DUS4
		Erasers	?
		Readers	?
Ψ	Non-canonical	Writers	PUS1 to PUS10, PUS7L, RPUSD1 to RPUSD4, DKC1
		Erasers	?
		Readers	PUM2?
Uridylation	Non-canonical	Writers	TUT4, TUT7
		Erasers	?
		Readers	DIS3L2
Nm	Canonical	Am Writer	FTSJ1 to FTSJ3, FBL
		Cm Writer	FTSJ1 to FTSJ3, CCDC76, FBL
		Gm Writer	TARBP1, FTSJ1, MRM1, RNMTL1, TrmH, FBL
		Um Writer	FTSJ1 to FTSJ3, FBL, TRMT44

**Table 2:** Position and type of RNA modifications that have been detected in human 18S rRNA.

Position	Modification	Position	Modification	Position	Modification	Position	Modification
27	Am	484	Am	822	Ψ	1328	Gm
34	Ψ	509	Gm	863	Ψ	1337	ac^4^C
36	Ψ	512	Am	866	Ψ	1347	Ψ
93	Ψ	517	Cm	867	Gm	1367	Ψ
99	Am	572	Ψ	897	Ψ	1383	Am
105	Ψ	576	Am	918	Ψ	1391	Cm
109	Ψ	590	Am	966	Ψ	1442	Um
116	Um	601	Gm	1004	Ψ	1445	Ψ
119	Ψ	609	Ψ	1031	Am	1447	Gm
121	Um	621	Cm	1045	Ψ	1490	Gm
159	Am	627	Um	1046	Ψ	1625	Ψ
166	Am	644	Gm	1056	Ψ	1639	m^7^G
172	Um	649	Ψ	1081	Ψ	1643	Ψ
174	Cm	651	Ψ	1136	Ψ	1668	Um
210	Ψ	668	Am	1174	Ψ	1678	Am
218	Ψ	681	Ψ	1177	Ψ	1692	Ψ
296	Ψ	683	Gm	1232	Ψ	1703	Cm
354	Um	686	Ψ	1238	Ψ	1804	Um
406	Ψ	797	Cm	1244	Ψ	1832	m^6^A
428	Um	799	Um	1248	m^1^acp^3^Ψ	1842	ac^4^C
436	Gm	801	Ψ	1272	Cm	1850	m^6^_2_A
462	Cm	814	Ψ	1288	Um	1851	m^6^_2_A
468	Am	815	Ψ	1326	Um		

**Table 3:** Position and type of RNA modifications that have been detected in human 28S rRNA.

Position	Modification	Position	Modification	Position	Modification	Position	Modification
389	Am	2495	Ψ	3804	Am	4417	m^5^C
391	Am	2619	Ψ	3809	Am	4426	Cm
1303	Gm	2774	Am	3820	Cm	4427	Ψ
1309	m^1^A	2791	Cm	3823	Ψ	4441	Ψ
1310	Am	2802	Am	3830	Ψ	4463	Ψ
1313	Am	2811	Cm	3832	Ψ	4464	Gm
1327	Cm	2824	Um	3846	Am	4468	Um
1509	Gm	2826	Ψ	3848	Cm	4469	Gm
1511	Am	2830	Ψ	3863	Ψ	4470	Ψ
1521	Am	2848	Cm	3866	Cm	4491	Ψ
1523	Ψ	2863	Gm	3878	Gm	4493	Am
1569	Ψ	3606	Gm	3899	Ψ	4500	m^3^U
1612	Gm	3616	Ψ	3904	Um	4502	Ψ
1664	Ψ	3618	Ψ	3923	Gm	4401	Ψ
1670	Ψ	3674	Ψ	3938	Ψ	4506	Cm
1731	Ψ	3680	Cm	4020	Gm	4522	Ψ
1747	Gm	3694	Ψ	4032	Cm	4541	Am
1760	Um	3697	Am	4166	Gm	4546	Ψ
1766	Ψ	3703	Am	4197	Um	4549	Ψ
1768	Ψ	3709	Ψ	4198	Gm	4560	Am
1769	Ψ	3713	Ψ	4220	m^6^A	4588	Gm
1779	Ψ	3723	Gm	4263	Ψ	4590	Um
1847	Ψ	3737	Ψ	4266	Ψ	4593	Gm
1849	Ψ	3739	Am	4269	Ψ	4598	Ψ
1858	Am	3741	Ψ	4276	Um	4606	Ψ
1868	Cm	3743	Ψ	4282	Ψ	4607	Gm
2338	Cm	3747	Ψ	4323	Ψ	4643	Ψ
2350	Am	3749	Ψ	4331	Ψ	4659	Ψ
2351	Gm	3761	m^5^C	4340	Gm	4937	Ψ
2352	Cm	3764	Am	4362	Gm	4966	Ψ
2388	Am	3771	Gm	4373	Ψ	4975	Ψ
2402	Um	3787	Cm	4390	Ψ	4506	Cm
2409	Cm	3797	Ψm	4393	Ψ		
2411	Gm	3801	Ψ	4412	Ψ		

**Table 4:** Regulatory roles of RNA modifications in various human diseases.

Modification	Physiological Role	Human Diseases	References
m^6^A	mRNA splicing, translation efficiency, transcriptional repression by lncRNAs	AML, CML, Obesity, Osteoporosis, hepatocellular carcinoma, ADHD, AD, PD	[21,153,154,155]
m^1^A	Translation efficiency, rRNA folding, tRNA folding and stability	Breast, ovarian, cervical, pancreatic and hepatocellular cancer, leukemia	[95,156,157,158]
A-to-I	Wobble codon recognition in tRNAs, mRNA stability and localization	Colorectal, gastric, esophageal and lung cancer, HCC	[159,160]
m^5^C	mRNA stability, translation efficiency, rRNA folding, tRNA folding and stability, lncRNA stability	Breast and bladder cancer, hypotonia, acidosis, ARID, DS	[161,162]
m^3^C	tRNA:mRNA stability, translation efficiency	Asthma, neoplastic and developmental pathologies	[163,164]
m^1^G	Reduces translation efficiency	Colorectal cancer	[165]
m^7^G	Translation regulators, tRNA stability	Lung cancer, HCC	[166,167]
Q	Protects tRNA from ribonuclease cleavage	Colorectal cancer, lymphoma	[130,168,169]
D	mRNA splicing, translation efficiency, tRNA structure	Lung cancer	[170]
Ψ	Ribosome assembly, translational efficiency, tRNA stability	Breast, prostate and lung cancer, HCC	[161]
Nm	Stability of RNAs	AD, asthma	[143]

## Data Availability

No new data was created or analyzed in this study. Data sharing is not applicable to this article.

## Supplementary Materials

The following supporting information can be downloaded at: https://www.mdpi.com/article/10.3390/ijms24032387/s1.
