# Supplementary material for: The Repertoire of RNA Modifications Orchestrates a Plethora of Cellular Responses
Source: Int J Mol Sci. 2023 Jan 25;24(3):2387. doi: 10.3390/ijms24032387 (PMC9916637; doi:10.3390/ijms24032387)
Supplement: Supplementary file 1 [file ijms-24-02387-s001.zip › ijms-2167798-supplementary.pdf]

International Journal of *Molecular Sciences*

MDPI

Review

The Repertoire of RNA Modifications Orchestrates a Plethora of Cellular Responses

Panagiotis G. Adamopoulos *, Konstantina Athanasopoulou, Glykeria N. Daneva and Andreas Scorilas

Department of Biochemistry and Molecular Biology, Faculty of Biology, National and Kapodistrian University of Athens, 15701 Athens, Greece; konnath@biol.uoa.gr (K.A.); gldaneva@biol.uoa.gr (G.N.D.);
ascorilas@biol.uoa.gr (A.S.)
* Correspondence: padamopoulos@biol.uoa.gr; Tel.: +30-210-72-74-686

Citation: Adamopoulos, P.G.; Athanasopoulou, K.; Daneva, G.N.; Scorilas, A. The Repertoire of RNA Modifications Orchestrates a Plethora of Cellular Responses. *Int. J. Mol. Sci.* **2023**, *24*, 2387. https://doi.org/ 10.3390/ijms24032387

Academic Editor: Demetrios A. Arvanitis

Received: 31 December 2022
Revised: 21 January 2023
Accepted: 23 January 2023
Published: 25 January 2023

Copyright: © 2023 by the authors. Submitted for possible open access publication under the terms and conditions of the Creative Commons Attribution (CC BY) license (https://creativecommons.org/licenses/by/4.0/).

Abstract: Although a plethora of DNA modifications have been extensively investigated in the last decade, recent breakthroughs in molecular biology, including high throughput sequencing techniques, have enabled the identification of post-transcriptional marks that decorate RNAs; hence, epitranscriptomics has arisen. This recent scientific field aims to decode the regulatory layer of the transcriptome and set the ground for the detection of modifications in ribose nucleotides. Until now, more than 170 RNA modifications have been reported in diverse types of RNA that contribute to various biological processes, such as RNA biogenesis, stability, and transcriptional and translational accuracy. However, dysfunctions in the RNA-modifying enzymes that regulate their dynamic level can lead to human diseases and cancer. The present review aims to highlight the epitranscriptomic landscape in human RNAs and match the catalytic proteins with the deposition or deletion of a specific mark. In the current review, the most abundant RNA modifications, such as N6-methyladenosine (m^6A), N5-methylcytosine (m^5C), pseudouridine (Ψ) and inosine (I), are thoroughly described, their functional and regulatory roles are discussed and their contributions to cellular homeostasis are stated. Ultimately, the involvement of the RNA modifications and their writers, erasers, and readers in human diseases and cancer is also discussed.

International Journal of *Molecular Sciences*

MDPI

Supplementary Table S1. Identified RNA modifications in different types of RNA from different organisms, based on the Modomics database.

Abbreviations	Full Name	Confirmed RNA type
ac^{6}A	N6-acetyladenosine	tRNA
Am	2'-O-methyladenosine	mRNA, rRNA, snRNA, snoRNA, tRNA
Ar(p)	2'-O-ribosyladenosine (phosphate)	tRNA
ct^{6}A	cyclic N6-threonylcarbamoyladenosine	tRNA
f^{6}A	N6-formyladenosine	mRNA
g^{6}A	N6-glycinylcarbamoyladenosine	tRNA
hm^{6}A	N6-hydroxymethyladenosine	mRNA
hn^{6}A	N6-hydroxynorvalylcarbamoyladenosine	tRNA
ht^{6}A	hydroxy-N6-threonylcarbamoyladenosine	tRNA
I	Inosine	mRNA, miRNA, tRNA
i^{6}A	N6-isopentenyladenosine	tRNA
Im	2'-O-methylinosine	mRNA, tRNA
io^{6}A	N6-(cis-hydroxyisopentenyl) adenosine	tRNA
m^{1}A	1-methyladenosine	mRNA, lncRNA, rRNA, tRNA
m^{1}Am	1,2'-O-dimethyladenosine	rRNA, tRNA
m^{1}I	1-methylinosine	tRNA
m^{1}Im	1,2'-O-dimethylinosine	tRNA
m^{2,8}A	2,8-dimethyladenosine	rRNA
m^{2}A	2-methyladenosine	rRNA, tRNA
m^6,^{6}A	N6, N6-dimethyladenosine	rRNA
m^6,^{6}Am	N6,N6,2'-O-trimethyladenosine	rRNA
m^{6}A	N6-methyladenosine	mRNA, miRNA, lncRNA, rRNA, snRNA, tRNA
m^{6}Am	N6,2'-O-dimethyladenosine	snRNA
m^{6}t^{6}A	N6-methyl-N6-threonylcarbamoyladenosine	tRNA
m^{8}A	8-methyladenosine	rRNA
ms^{2}ct^{6}A	2-methylthio cyclic N6-threonylcarbamoyladenosine	tRNA
ms^{2}hn^{6}A	2-methylthio-N6-hydroxynorvalylcarbamoyladenosine	tRNA
ms^{2}i^{6}A	2-methylthio-N6-isopentenyladenosine	tRNA
ms^{2}io^{6}A	2-methylthio-N6-(cis-hydroxyisopentenyl) adenosine	tRNA
ms^{2}m^{6}A	2-methylthio-N6-methyladenosine	tRNA
ms^{2}t^{6}A	2-methylthio-N6-threonylcarbamoyladenosine	tRNA
msms^{2}i^{6}A	2- methylthiomethylenethio-N6-isopentenyl-adenosine	tRNA
pA	adenosine-5'-monophosphate	
pA2'3'cp	adenosine-5'-phosphate-2',3'-cyclic phosphate	
pac^{6}A	N6-acetyladenosine-5'-monophospate	
pAm	2'-O-methyladenosine 5'-(dihydrogen phosphate)	
pAp	adenosine-3',5'-diphosphate	
pAr(p)	2'-O-[(5'-phospho)ribosyl]adenosine-5'-monophosphate	

International Journal of Molecular Sciences

MDPI

pct^{6}A	cyclic N6-threonylcarbamoyladenosine-5'-monophosphate	
pf^{6}A	N6-formyladenosine-5'-monophospate	
pg^{6}A	N6-glycinylcarbamoyladenosine-5'-monophosphate	
phm^{6}A	N6-hydroxymethyladenosine-5'-monophosphate	
phn^{6}A	N6-hydroxynorvalylcarbamoyladenosine-5'-monophosphate	
pht^{6}A	hydroxy-N6-threonylcarbamoyladenosine-5'-monophosphate	
pI	inosine-5'-monophosphate	
pi^{6}A	N6-isopentenyl-adenosine-5'-monophosphate	
pIm	2'-O-methylinosine-5'-monophosphate	
pio^{6}A	N6-(cis-hydroxyisopentenyl)adenosine-5'-monophospate	
pm^{1}A	6-hydro-1-methyladenosine-5'-monophosphate	
pm^{1}Am	1,2'-O-dimethyladenosine-5'-monophosphate	
pm^{1}I	1-methylinosine-5'-monophosphate	
pm^{1}Im	1,2'-O-dimethylinosine-5'-monophosphate	
pm^{2,8}A	2,8-dimethyladenosine-5'-monophosphate	
pm^{2}A	2-methyladenosine-5'-monophosphate	
pm^{6,6}A	6N-dimethyladenosine-5'-monophosphate	
pm^{6,6}Am	N6,N6,2'-O-trimethyladenosine-5'-monophospate	
pm^{6}A	N6-methyladenosine-5'-monophosphate	
pm^{6}Am	N6,2'-O-dimethyladenosine-5'-monophospate	
pm^{6}t^{6}A	N6-methyl-N6-threonylcarbamoyladenosine-5'-monophosphate	
pm^{8}A	8-methyladenosine-5'-monophosphate	
pms^{2}ct^{6}A	2-methylthio cyclic N6-threonylcarbamoyladenosine-5'-monophosphate	
pms^{2}hn^{6}A	2-methylthio-N6-hydroxynorvalylcarbamoyladenosine-5'-monophosphate	
pms^{2}i^{6}A	2-methylthio-N6-isopentenyl-adenosine-5'-monophosphate	
pms^{2}io^{6}A	2-methylthio-N6-(cis-hydroxyisopentenyl) adenosine-5'-monophosphate	
pms^{2}m^{6}A	2-methylthio-N6-methyladenosine-5'-monophosphate	
pms^{2}t^{6}A	2-methylthio-N6-threonylcarbamoyladenosine-5'-monophosphate	
pmsms^{2}i^{6}A	2- methylthiomethylenethio-N6-isopentenyl-adenosine-5'-monophosphate	
pppA	adenosine-5'-triphosphate	
pt^{6}A	N6-threonylcarbamoyladenosine-5'-monophosphate	
t^{6}A	N6-threonylcarbamoyladenosine	tRNA
xA	unknown modified adenosine	rRNA, tRNA
ac^{4}C	N4-acetylcytidine	mRNA, rRNA, tRNA
ac^{4}Cm	N4-acetyl-2'-O-methylcytidine	
C+	agmatidine	tRNA
Cm	2'-O-methylcytidine	mRNA, rRNA, snRNA, tRNA
f^{5}C	5-formylcytidine	tRNA
f^{5}Cm	5-formyl-2'-O-methylcytidine	tRNA
hm^{5}C	5-hydroxymethylcytidine	mRNA, rRNA, tRNA

International Journal of Molecular Sciences MDPI

hm^{5}Cm	2'-O-methyl-5-hydroxymethylcytidine	snRNA, tRNA
ho^{5}C	5-hydroxycytidine	rRNA
k^{2}C	2-lysidine	tRNA
m^{3}C	3-methylcytidine	tRNA
m^{4,4}C	N4, N4-dimethylcytidine	
m^{4,4}Cm	N4, N4,2'-O-trimethylcytidine	
m^{4}C	N4-methylcytidine	rRNA
m^{4}Cm	N4,2'-O-dimethylcytidine	rRNA
m^{5}C	5-methylcytidine	mRNA, miRNA, lncRNA, rRNA, snRNA, snoRNA, tRNA
m^{5}Cm	5,2'-O-dimethylcytidine	mRNA, rRNA, tRNA
pac^{4}C	N(4)-acetylcytidine-5'-monophosphate	
pac^{4}Cm	[(2~{R},3~{R},4~{R},5~{R})-5-(4-acetamido-2-oxidanylidene-pyrimidin-1-yl)-4-methoxy-3-oxidanyl-oxolan-2-yl]methyl dihydrogen phosphate	
pC	cytidine-5'-monophosphate	
pC+	agmatidine-5'-monophosphate	
pC2'3'cp	cytidine-5'-phosphate-2',3'-cyclic phosphate	
pCm	O2'-methylcytidine-5'-monophosphate	
pf^{5}C	5-formylcytidine 5'-(dihydrogen phosphate)	
pf^{5}Cm	5-formyl-2'-O-methylcytidine-5'-monophosphate	
phm^{5}C	5-(hydroxymethyl)cytidine 5'-(dihydrogen phosphate)	
phm^{5}Cm	2'-O-methyl-5-hydroxymethylcytidine-5'-monophosphate	
pho^{5}C	5-hydroxycytidine-5'-monophosphate	
pk^{2}C	2-lysidine-5'-monophosphate	
pm^{3}C	3-Methylcytidine- 5'-monophosphate	
pm^{4,4}C	[(2~{R},3~{S},4~{R},5~{R})-5-[4-(dimethylamino)-2-oxidanylidene-pyrimidin-1-yl]-3,4-bis(oxidanyl)oxolan-2-yl]methyl dihydrogen phosphate	
pm^{4,4}Cm	N4,N4,2'-O-trimethylcytidine-5'-monophospate	
pm^{4}C	4-methyl, cytidine-5'-monophosphate	
pm^{4}Cm	4N,O2'-methylcytidine-5'-monophosphate	
pm^{5}C	5-methylcytidine-5'-monophosphate	
pm^{5}Cm	5,2'-O-dimethylcytidine-5'-monophosphate	
ps^{2}C	2-thiocytidine-5'-monophosphate	
s^{2}C	2-thiocytidine	tRNA
xC	unknown modified cytidine	rRNA, tRNA
G+	archaeosine	tRNA
galQ	galactosyl-queuosine	tRNA
gluQ	glutamyl-queuosine	tRNA
Gm	2'-O-methylguanosine	mRNA, rRNA, snRNA, tRNA
Gr(p)	2'-O-ribosylguanosine (phosphate)	
imG	wyosine	tRNA
imG-14	4-demethylwyosine	
imG2	isowyosine	

$m^{1}G$	1-methylguanosine	mRNA, rRNA, tRNA
$m^{1}Gm$	1,2'-O-dimethylguanosine	
$m^{2,2,7}G$	N2,N2,7-trimethylguanosine	
$m^{2,2}G$	N2,N2-dimethylguanosine	mRNA, rRNA, tRNA
$m^{2,2}Gm$	N2,N2,2'-O-trimethylguanosine	
$m^{2,7}G$	N2,7-dimethylguanosine	
$m^{2,7}Gm$	N2,7,2'-O-trimethylguanosine	
$m^{2}G$	N2-methylguanosine	rRNA, snRNA, tRNA
$m^{2}Gm$	N2,2'-O-dimethylguanosine	
$m^{7}G$	7-methylguanosine	mRNA, miRNA, rRNA, tRNA
manQ	mannosyl-queuosine	tRNA
mimG	methylwyosine	
o2yW	peroxywybutosine	tRNA
OHyW	hydroxywybutosine	
OHyWx	undermodified hydroxywybutosine	
OHyWy	methylated undermodified hydroxywybutosine	
oQ	epoxyqueuosine	
pG	guanosine-5'-monophosphate	
pG+	archaeosine-5'-monophosphate	
pG2'3'cp	guanoside-5'-phosphate-2',3'-cyclic phosphate	
pgalQ	galactosyl-queuosine-5'-monophosphate	
pgluQ	glutamyl-queuosine-5'-monophosphate	
pGm	O2'-methylguanosine-5'-monophosphate	
pGp	guanosine-3',5'-diphosphate	
pGr(p)	2'-O-ribosylguanosine (phosphate)-5'-monophosphate	
pimG	wyosine-5'-monophospate	
pimG-14	4-demethylwyosine-5'-monophosphate	
pimG2	isowyosine-5'-monophosphate	
$pm^{1}G$	1N-methylguanosine-5'-monophosphate	
$pm^{1}Gm$	1,2'-O-dimethylguanosine-5'-monophosphate	
$pm^{2,2,7}G$	N2,N2,7-trimethylguanosine-5'-monophosphate	
$pm^{2,2}G$	N2-dimethylguanosine-5'-monophosphate	
$pm^{2,2}Gm$	N2,N2,2'-O-trimethylguanosine-5'-monophospate	
$pm^{2,7}G$	N2,7-dimethylguanosine-5'-monophosphate	
$pm^{2,7}Gm$	N2,7,2'-O-trimethylguanosine-5'-monophosphate	
$pm^{2}G$	2N-methylguanosine-5'-monophosphate	
$pm^{2}Gm$	N2,2'-O-dimethylguanosine-5'-monophospate	
$pm^{7}G$	7N-methyl-8-hydroguanosine-5'-monophosphate	
pmanQ	mannosyl-queuosine-5'-monophosphate	
pmimG	methylwyosine-5'-monophosphate	
pOHyW	hydroxywybutosine-5'-monophosphate	
pOHyWx	undermodified hydroxywybutosine-5'-monophospate	
pOHyWy	methylated undermodified hydroxywybutosine-5'-monophosphate	

International Journal of Molecular Sciences

poQ	epoxyqueuosine-5'-monophosphate	
ppG	guanosine-5'-diphosphate	
pppG	guanosine-5'-triphosphate	
ppreQ0	7-cyano-7-deazaguanosine-5'-monophosphate	
ppreQ1	7-aminomethyl-7-deazaguanosine-5'-monophosphate	
pQ	queuosine-5'-monophospate	
preQ0	7-cyano-7-deazaguanosine	
preQ0base	7-cyano-7-carbaguanine	
preQ1	7-aminomethyl-7-deazaguanosine	tRNA
preQ1base	7-aminomethyl-7-carbaguanine	
pyW	wybutosine-5'-monophosphate	
pyW-58	7-aminocarboxypropylwyosine methyl ester-5'-monophosphate	
pyW-72	7-aminocarboxypropylwyosine-5'-monophosphate	
pyW-86	7-aminocarboxypropyl-demethylwyosine-5'-monophosphate	
Q	queuosine	tRNA
Qbase	queuine	
xG	unknown modified guanosine	rRNA, tRNA
yW	wybutosine	rRNA, tRNA
yW-58	7-aminocarboxypropylwyosine methyl ester	
yW-72	7-aminocarboxypropylwyosine	
yW-86	7-aminocarboxypropyl-demethylwyosine	
acp^3D	3-(3-amino-3-carboxypropyl)-5,6-dihydrouridine	tRNA
acp^3U	3-(3-amino-3-carboxypropyl)uridine	rRNA, tRNA
$acp^3\Psi$	3-(3-amino-3-carboxypropyl)pseudouridine	
chm^5U	5-carboxyhydroxymethyluridine	
cm^5s^2U	5-carboxymethyl-2-thiouridine	
cm^5U	5-carboxymethyluridine	
$cmnm^5ges^2U$	5-carboxymethylaminomethyl-2-geranylthiouridine	tRNA
$cmnm^5s^2U$	5-carboxymethylaminomethyl-2-thiouridine	tRNA
$cmnm^5se^2U$	5-carboxymethylaminomethyl-2-selenouridine	
$cmnm^5U$	5-carboxymethylaminomethyluridine	tRNA
$cmnm^5Um$	5-carboxymethylaminomethyl-2'-O-methyluridine	tRNA
cmo^5U	uridine 5-oxyacetic acid	tRNA
cnm^5U	5-cyanomethyluridine	
D	dihydrouridine	rRNA, tRNA
ges^2U	2-geranylthiouridine	
ho^5U	5-hydroxyuridine	
inm^5s^2U	5-(isopentenylaminomethyl)-2-thiouridine	
inm^5U	5-(isopentenylaminomethyl)uridine	
inm^5Um	5-(isopentenylaminomethyl)-2'-O-methyluridine	
$m^1acp^3\Psi$	1-methyl-3-(3-amino-3-carboxypropyl)pseudouridine	rRNA
$m^1\Psi$	1-methylpseudouridine	rRNA, tRNA
m^3U	3-methyluridine	rRNA, tRNA
m^3Um	3,2'-O-dimethyluridine	

International Journal of Molecular Sciences

MDPI

m^{3}Ψ	3-methylpseudouridine	rRNA
m^{5}D	5-methyldihydrouridine	
m^{5}s^{2}U	5-methyl-2-thiouridine	tRNA
m^{5}U	5-methyluridine	rRNA, tRNA
m^{5}Um	5,2'-O-dimethyluridine	tRNA
mchm^{5}U	5-(carboxyhydroxymethyl)uridine methyl ester	tRNA
mchm^{5}Um	5-(carboxyhydroxymethyl)-2'-O-methyluridine methyl ester	
mcm^{5}s^{2}U	5-methoxycarbonylmethyl-2-thiouridine	tRNA
mcm^{5}U	5-methoxycarbonylmethyluridine	tRNA
mcm^{5}Um	5-methoxycarbonylmethyl-2'-O-methyluridine	tRNA
mcmo^{5}U	uridine 5-oxyacetic acid methyl ester	tRNA
mcmo^{5}Um	2'-O-methyluridine 5-oxyacetic acid methyl ester	
mnm^{5}ges^{2}U	5-methylaminomethyl-2-geranylthiouridine	tRNA
mnm^{5}s^{2}U	5-methylaminomethyl-2-thiouridine	tRNA
mnm^{5}se^{2}U	5-methylaminomethyl-2-selenouridine	tRNA
mnm^{5}U	5-methylaminomethyluridine	tRNA
mo^{5}U	5-methoxyuridine	tRNA
nchm^{5}U	5-carbamoylhydroxymethyluridine	
ncm^{5}s^{2}U	5-carbamoylmethyl-2-thiouridine	
ncm^{5}U	5-carbamoylmethyluridine	tRNA
ncm^{5}Um	5-carbamoylmethyl-2'-O-methyluridine	
nm^{5}ges^{2}U	5-aminomethyl-2-geranylthiouridine	
nm^{5}s^{2}U	5-aminomethyl-2-thiouridine	
nm^{5}se^{2}U	5-aminomethyl-2-selenouridine	
nm^{5}U	5-aminomethyluridine	
pacp^{3}D	3-(3-amino-3-carboxypropyl)-5,6-dihydrouridine-5'-monophosphate	
pacp^{3}U	3-(3-amino-3-carboxypropyl)uridine-5'-monophosphate	
pchm^{5}U	5-carboxyhydroxymethyluridine-5'-monophosphate	
pcm^{5}s^{2}U	5-carboxymethyl-2-thiouridine-5'-monophosphate	
pcm^{5}U	5-carboxymethyluridine-5'-monophosphate	
pcmnm^{5}ges^{2}U	5-carboxymethylaminomethyl-2-geranylthiouridine-5'-monophosphate	
pcmnm^{5}s^{2}U	5-carboxymethylaminomethyl-2-thiouridine-5'-monophosphate	
pcmnm^{5}se^{2}U	5-carboxymethylaminomethyl-2-selenouridine-5'-monophosphate	
pcmnm^{5}U	5-carboxymethylaminomethyluridine-5'-monophosphate	
pcmnm^{5}Um	5-carboxymethylaminomethyl-2'-O-methyluridine-5'-monophosphate	
pcmo^{5}U	5-(carboxymethoxy) uridine-5'-monophosphate	
pcnm^{5}U	5-cyanomethyluridine-5'-monophosphate	
pD	5,6-dihydrouridine-5'-monophosphate	
pges^{2}U	2-geranylthiouridine-5'-monophosphate	
pho^{5}U	5-hydroxyuridine-5'-monophosphate	
pinm^{5}s^{2}U	5-(isopentenylaminomethyl)-2-thiouridine-5'-monophosphate	

International Journal of Molecular Sciences

MDPI

pinm5U	5-(isopentenylaminomethyl)uridine-5'-monophosphate	
pinm5Um	5-(isopentenylaminomethyl)-2'-O-methyluridine-5'-monophosphate	
pm1acp3Ψ	1-methyl-3-(3-amino-3-carboxypropyl)pseudouridine-5'-monophosphate	
pm1Ψ	1-methylpseudouridine-5'-monophosphate	
pm3U	3-methyluridine-5'-monophosphate	
pm3Um	3,2'-O-dimethyluridine-5'-monophosphate	
pm3Ψ	3-methylpseudouridine-5'-monophosphate	
pm5D	5-methyldihydrouridine-5'-monophosphate	
m5D	5-methyldihydrouridine	
ps4U	4-thiouridine-5'-monophosphate	
s4U	4-thiouridine	tRNA
pD	5,6-dihydrouridine-5'-monophosphate	
pΨ	pseudouridine-5'-monophosphate	
Ψ	pseudouridine	mRNA, miRNA, rRNA, snRNA, snoRNA, tRNA
?U	unknown modified uridine	rRNA, snRNA, tRNA
U	uridine	rRNA, tRNA
